# Assessment of Health-Related Quality of Life and Its Associated Factors Among Cardiovascular Disease Patients at a Teaching Hospital in Northwest Ethiopia: A Cross-Sectional Study

**DOI:** 10.1155/bmri/1159456

**Published:** 2025-03-07

**Authors:** Assefa Belay Asrie, Mulugeta Dereje, Amanuel Getachew, Betelhem Genetu

**Affiliations:** ^1^Department of Pharmacology, School of Pharmacy, College of Medicine and Health Sciences, University of Gondar, Gondar, Ethiopia; ^2^School of Pharmacy, College of Medicine and Health Sciences, University of Gondar, Gondar, Ethiopia

**Keywords:** cardiovascular diseases, determinants of health-related quality of life, EQ-5D five-level questionnaire, Ethiopia, health-related quality of life, utility index value, VAS score, visual analog scale

## Abstract

**Background:** Health-related quality of life (HRQoL) has become a widely recognized outcome measure to assess the impact of illnesses or effectiveness of treatments. This study was carried out to investigate HRQoL and associated factors among cardiovascular disease patients.

**Method:** This is a cross-sectional study and was carried out from July 01 to August 30, 2021. The patients were recruited using systematic random sampling technique and data was collected using EQ-5D five-level (EQ-5D-5L) questionnaires and EQ visual analog scale (EQ VAS). Utility index values were calculated using disutility weights set in Ethiopian context. Mann–Whitney *U* and Kruskal–Wallis tests were employed to compare the median index values and EQ VAS scores across subgroups. Tobit regression analysis was performed to determine factors associated with HRQoL.

**Results:** Performing usual activities (76.8%) and pain/discomfort (74.9%) were the first and the second dimensions of most frequently reported health problems, respectively. The overall median (interquartile range) EQ-5D-5L index value and VAS score were 0.82 (0.65–0.92) and 70.0 (60.0–80.0), respectively. Older age, multiple CVD diagnoses, and adherence problems to medications were found to be negatively associated with HRQoL.

**Conclusion:** In conclusion, performing usual activities and pain/discomfort were the dimensions with the most frequently reported problems. This finding dictates the importance of giving special attention to these dimensions in managing CVD patients. Besides, older age, multiple CVDs, and nonadherence to medications were negatively associated with HRQoL. Thus, acting in consideration of these factors in patient management may have positive implications in improving their HRQoL.

## 1. Background

Cardiovascular diseases (CVDs) are prevalent, and the global incidences are rising. From 1990 to 2019, the number of CVD deaths increased steadily from 12.1 to 18.6 million, and the prevalence of all CVD cases was nearly doubled worldwide [1]. A WHO report showed that CVDs are the largest contributor to the total noncommunicable disease burden in African countries [2]. According to other reports, CVDs account for 38.3% of fatalities linked to noncommunicable diseases, making them the leading cause of noncommunicable disease burden overall, particularly in sub-Saharan Africa [3, 4]. In contrast to a number of high-income countries which recorded reductions in cardiovascular deaths [5], a nearly 50% increase in the CVD burden was recorded in Africa over the past three decades [6]. A meta-analysis and systematic review revealed that the prevalence of CVDs ranges from 1% to 20% in Ethiopia, with a pooled prevalence of 5% [7]. Additionally, a different report indicated a significant contribution of these diseases to the increasing healthcare costs of the country [8]. Furthermore, another study revealed that CVDs are among the top causes of death in the country. The study also reported that the burden of CVDs in Ethiopia is high and has been increasing in older age groups, and the findings urge the country to consider CVDs as a priority public health problem [9].

CVDs pose a substantial burden on patients' health-related quality of life (HRQoL) and can affect their ability to function [10, 11]. HRQoL is an indicator of how a health condition and its treatment affect physical, emotional, and social well-being [12]. In light of this, HRQoL has become a widely recognized outcome measure in the assessment of the impact of illness or the effectiveness of treatments [13, 14]. HRQoL is currently considered an important patient-reported health outcome measure in patients with CVDs. Assessing the impact of clinical managements on the patients' quality of life in addition to the usual clinical outcomes is vital [15]. Such investigations on HRQoL can point out the patients' perspective on their own health [16] and be utilized as a reliable indicator of unmet needs of the patients and the effectiveness of clinical interventions [17]. In support of this, the American Heart Association recommends that the inclusion of patients' HRQoL is an important measure of cardiovascular health among patients with CVD [18]. Many generic and disease-specific HRQoL measurement tools have been devised for patients with CVDs [19–21]. The EQ-5D tool is a convenient and user-friendly instrument for assessing HRQoL [22] and has been validated in the Ethiopian context and the disutility scores determined [23].

Besides, given the impact of CVDs on HRQoL [24, 25], there has been an emphasis on researching the factors influencing HRQoL in CVD patients [24, 26, 27]. A number of factors have been shown to have considerable association with HRQoL. Depression and disability are the well-known of them [28, 29]. Others such as age, social support, comorbid conditions [30], residence, educational status [31], and patient level of engagement in self-care have been also demonstrated to be significantly associated with HRQoL [32]. Studies also showed that adherence to medications has a positive correlation with HRQoL in patients with different CVDs. One study revealed poor self-reported medication adherence is associated with a decline in HRQoL in older adults with hypertension [12]. A study entitled “Adherence to Therapy and Quality of Life in Hypertensive Patients” has also revealed a statistically significant positive relationship between medication adherence and quality of life [33]. Another study indicated that lower HRQoL was associated with nonadherence among heart failure patients [34]. From literature searches, however, studies dedicated to HRQoL and determinant factors, including adherence among others, in patients with CVD as a group are lacking.

As it can be seen from the literature reviews, CVDs are a worldwide concern and the leading cause of death in Ethiopia. Also, CVDs compromise the patients' HRQoL together with other determinant factors, including adherence to medications and other patient-related factors. However, data on HRQoL and determinants of HRQoL among patients with CVDs are limited in Ethiopian settings. Thus, this study was carried out to investigate the HRQoL and determinant factors among patients with CVDs. The findings of this study may give useful insight about the important factors to consider in the management of CVDs and the overall health status of the patients receiving healthcare services at the study site.

## 2. Methods

### 2.1. Study Setting

The study was conducted at University of Gondar Comprehensive Specialized Hospital (UoGCSH). The hospital is located in Gondar Town, Amhara National Regional State, Ethiopia, and is about 738 km northwest of Addis Ababa, the capital city of the country. The hospital is the only referral, teaching, and research center in Central Gondar Zone, Northwest Ethiopia, and contributes to generating competent health professionals to the country. It provides health services for more than 13 million people in the Central Gondar and neighboring zones. It has 28 wards with about 960 beds and 15 outpatient service areas in different departments. Every year, around 400,000 patients visit the outpatient departments and more than 30,000 admission cases are seen. The study was conducted at the chronic illness outpatient clinic, which is one of the many outpatient clinics in the hospital. The clinic provides services for patients with different chronic illnesses, including CVDs.

### 2.2. Study Design and Period

Hospital-based cross-sectional study design was employed to assess HRQoL among CVD patients. The study was conducted from July 01 to August 30, 2021.

### 2.3. Source and Study Population

#### 2.3.1. Source Population

The patients with CVD who were on follow-up care at the chronic illness outpatient clinic of the hospital are the source population of the study.

#### 2.3.2. Sample Population

The sample population is the subset of the source population chosen based on the sampling technique and inclusion criteria and participated in the study.

### 2.4. Inclusion and Exclusion Criteria

#### 2.4.1. Inclusion Criteria

• Patients diagnosed with CVD
• Age 18 years or older
• Patients who were on follow-up care at the chronic illness outpatient clinic of the hospital at least for 3 months

#### 2.4.2. Exclusion Criteria

• Patients with established psychiatric illnesses
• Patients who did not give consent to participate in the study

### 2.5. Sample Size Determination and Sampling Technique

Single population proportion formula was used for sample determination with the assumption of *Z* value 1.96 with 95% confidence interval, 5% margin of error, and 50% proportion of patients with CVDs are in poor quality of life.

 $$\begin{array}{c} n=\frac{Z^{2}p\left(1-p\right)}{w^{2}}=\frac{{\left(1.96\right)}^{2}0.5\left(1-0.5\right)}{{\left(0.05\right)}^{2}}=384.16\approx 384. \end{array}$$

To the sample found using the above formula, 10% contingency was added, and then, a total of 422 patients were considered to be included in the study.

As of the record (log book) in the chronic illness follow-up clinic, about 20 CVD patients visit the clinic every working day. Therefore, every other patient attending the clinic during the data collection period was selected for the study.

### 2.6. Data Collection Instruments, Process, and Quality Assurance

Sociodemographic details of the participants were collected using questions adapted from previous studies. Data on adherence to medications was collected by directly inquiring the patients to rate their own level of adherence as high, moderate, or low. Data pertaining to HRQoL were collected using the EQ-5D five-level (EQ-5D-5L) questionnaires and EQ visual analog scale (EQ VAS) developed by the EuroQol group. It is a generic instrument that consists of five dimensions with five levels of descriptive system questionnaires and the visual analog scale, which is used for subjective assessment of one's current health state from the patient's perspective. The five dimensions orderly are mobility, self-care, usual activities, pain/discomfort, and anxiety/depression. The EQ VAS gives a way for individuals to rate their own overall current health [21]. Disutility coefficients (decrement in utility) in each dimension have been determined in the Ethiopian general population using a hybrid regression model [23]. The data was collected by three graduating pharmacy students. To ensure the quality of the data, the data collectors were trained on the overall study design and objectives, the contents of the questionnaire, and approaching interviewees. Furthermore, each filled-out questionnaire was checked for completeness and clarity upon receiving, and some questionnaires with inconsistent data were excluded from the study.

### 2.7. Study Variables

#### 2.7.1. Dependent Variable

• HRQoL (EQ-5D-5L index value and EQ-VAS score)

#### 2.7.2. Independent Variables

• Sociodemographic characteristics (sex, age, marital status, educational level, occupation, and residence)
• Clinical characteristics (number of CVDs, time since CVD diagnosed, comorbidity, and adherence to medications)

### 2.8. Operational Definitions

#### 2.8.1. CVDs

CVDs are a group of medical problems that affect the heart and blood vessels including, but not restricted to, heart failure, hypertension, arrhythmias, cardiomyopathy, valvular heart disease, coronary artery disease, and associated cerebrovascular accidents such as stroke and myocardial infarction.

#### 2.8.2. Multiple CVD Conditions

This pertains to diagnosis of two or more CVDs per an individual patient.

#### 2.8.3. Comorbidities

Comorbidities are clinical conditions other than CVDs, for example, diabetes mellitus and rheumatoid arthritis.

#### 2.8.4. HRQoL

HRQoL pertains to patients' self-perception about their own health reflected by EQ-5D-5L index values and EQ VAS scores, higher values or scores showing better HRQoL.

#### 2.8.5. High Adherence to Medications

This pertains to patient self-rating of own adherence level based on self-judgment of their experience of missing doses for any reason.

#### 2.8.6. Nonadherence to Medications

Patient self-rating of moderate or low adherence to their medications based on experience of missing doses for any reason was regarded as nonadherence.

### 2.9. Data Analysis and Interpretation

The level of adherence was measured by directly inquiring the participants to rate their level of adherence to their medications as high, moderate, or low. Moderate and low adherence levels are referred to as nonadherence in our discussion onwards. Each dimension of the EQ-5D-5L descriptive system was assigned a severity level with 5 levels, 1 representing no problem and 5 representing extreme problem or inability. The EQ-5D-5L index values were computed using disutility coefficients (reductions in utility index values from 1 with each level of compromise in each dimension). The reductions in utility index values with each level of compromise in each dimension were previously determined in Ethiopian general population using a hybrid regression model [23]. A respondent with utility index value equal to 1 was considered with no problem in all dimensions and taken as a reference from which decrement coefficients in any dimension would be deducted. The deduction value in each dimension increases as we move from Level 2 to Level 5. Level 1 response would not contribute any reduction from the prefect health condition (utility index value 1). The equation was set in an Excel sheet, and as the data were copied from SPSS to the Excel sheet, utility index value was automatically calculated for each patient. To illustrate, a respondent with slight problems in doing his/her usual activities and moderately anxious and no problem (Level 1 response) in the other three dimensions would have utility index value of 1 − (0.0323013 + 0.0848133) = 0.88.

In the Shapiro–Wilk test, EQ-5D-5L utility index values and EQ VAS scores were not found to be normally distributed across subgroups (*p* < 0.05), and hence, we compared the median (interquartile ranges, IQRs) between subgroups. The differences in median EQ-5D-5L utility index values and EQ VAS scores between subgroups were assessed using Mann–Whitney *U* and Kruskal–Wallis tests. In the face of this, however, we calculated the overall mean and median values for comparison with the previous overall results of previous studies. Additionally, we censored the utility index value at 1 and the EQ VAS score at 100, and multivariate Tobit regression analysis was performed to examine the determinants of HRQoL. Statistical analyses were performed using STATA Version 17 and SPSS Version 26. Statistical differences or associations were considered significant at *p* < 0.05.

### 2.10. Ethical Consideration

The study proposal was approved by the Ethical Review Committee of the School of Pharmacy, University of Gondar, and an ethical approval letter was obtained from the School of Pharmacy, on behalf of the committee (Ref. Number SOP/262/2013). In addition, official permission was obtained from the hospital's administrative office to formalize the data collection. Each participant was included in the study after being informed about the purpose of the study and agreeing to be interviewed. Moreover, the data collected was kept confidential and no data relating to personal identification was collected.

## 3. Results

### 3.1. Sociodemographic and Some Clinical Characteristics of the Respondents

The proportions of the male and female participants were 50.2% and 49.8%, respectively. Of all the participants, 51.2% were 18–50 years old, while 48.8% of them were ≥ 51 years old. In their marital status, the majority of them were married (61.8%), and in educational level, more than half of them were illiterate (59.2%). The highest proportions of the participants were housewives (31.1%). In terms of residence, most of them lived in urban areas (66.4%). Of the respondents surveyed, 67.1% had only one CVD diagnosis. About 66.6% of the participants were with ≤ 5 years since their CVDs were diagnosed. Less than half of the patients (41.2%) had non-CVS comorbidities. In the direct inquiry to rate their level of adherence, 61.4%, 31.5%, and 7.1% of the patients rated that they had high, moderate, and low levels of adherence to their medications, respectively (Table 1).

### 3.2. HRQoL of CVD Patients

#### 3.2.1. Responses to EQ-5D-5L

**Figure 1:**
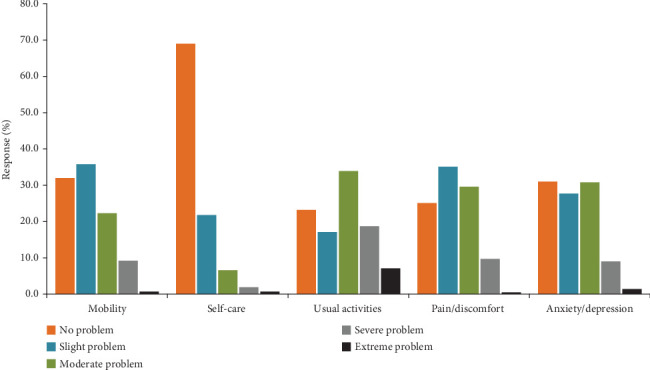
Proportions of responses by level of severity for EQ-5D-5L dimensions in CVD patients attending the outpatient chronic illness clinic at UoGCSH, Northwest Ethiopia, 2021 (*n* = 422).

Figure 1 displays the percentage distributions of the responses to the EQ-5D-5L questionnaires. Difficulty performing usual activities was the most commonly reported health issue, with 76.8% of the participants reported slight difficulty to complete inability to do their routine activities. Pain/discomfort was the second most commonly reported health problem, with 74.9% of patients reported slight to severe pain. Conversely, ability to self-care was the least common problem, with 31.0% of patients reported mild difficulty to inability to wash and dress themselves. Of all the participants, 13.5% reported that they had no problem in any of the five dimensions (scored a utility index value of 1). On the VAS scale, only 8.8% of the patients reported a score of 90 or 95, whereas none of them reported the best level of health status (100).

#### 3.2.2. EQ-5D-5L Index Value and EQ-VAS Score

The median (IQR) EQ-5D-5L utility index values and EQ VAS scores were 0.82 (0.65–0.92) and 70.0 (60.0–80.0), respectively, while the mean (SD) EQ-5D-5L index values and EQ VAS scores were 0.74 (0.26) and 68.0 (15.4), respectively. The median EQ-5D-5L index value (*p* < 0.05) and EQ VAS score (*p* ≤ 0.01) of patients older than 50 years were significantly lower compared with the values of those aged 18–50 years. The median EQ-5D-5L index value (*p* < 0.05) and EQ VAS score (*p* ≤ 0.01) of the married group were significantly greater compared to those of the single group. The median EQ-5D-5L index value was significantly greater in patients with tertiary education compared to patients who were illiterate and/or with a secondary education level (*p* ≤ 0.01). The median EQ-5D-5L index value was significantly higher in government employees and traders compared to farmers (*p* < 0.05). The median EQ VAS score of traders was significantly greater (*p* < 0.05) compared to that of government employees, farmers, or others. The median EQ-5D-5L index value (*p* ≤ 0.01) and EQ VAS score (*p* ≤ 0.01) were significantly higher in patients with only one CVD diagnosis compared to those with two or more diagnoses. Compared to rural residents, patients from urban areas had significantly higher median EQ-5D-5L index value and EQ VAS score (*p* ≤ 0.01). Furthermore, the median EQ-5D-5L index value and EQ VAS score were significantly higher (*p* ≤ 0.01) in patients with high adherence to their medications compared to patients with a moderate or low level of adherence. On the other hand, there were no significant differences in the median EQ-5D-5L index values and EQ VAS scores with sex, time since diagnosis of CVDs, and non-CVS comorbidities (Table 2).

#### 3.2.3. Determinants of HRQoL

In Tobit regression analysis, older age (*β* = −0.133, 95% CI: −0.243, −0.023, *p* < 0.05), having two or more CVDs (*β* = −0.121, 95% CI: −0.174, −0.068, *p* ≤ 0.01), and moderate (*β* = −0.142, 95% CI: −0.193, −0.091, *p* ≤ 0.01) or low (*β* = −0.256, 95% CI: −0.332, −0.180, *p* ≤ 0.01) adherence to medications were significantly negatively associated with EQ-5L-5D index value. Similarly, older age (*β* = −4.313, 95% CI: −7.206, −1.420, *p* ≤ 0.01), having two more CVDs (*β* = −3.682, 95% CI: −6.544, −0.823, *p* < 0.05), and moderate (*β* = −8.921, 95% CI: −11.853, −5.989, *p* ≤ 0.01) or low (*β* = −19.610, 95% CI: −24.761, −14.459, *p* ≤ 0.01) adherence were shown to have significant negative association with EQ VAS score (Table 3).

## 4. Discussion

HRQoL assessments help outline patients' perspective on their own health [16] and are used to evaluate the effects of diseases, complications, and treatments on their health status [35, 36]. This study was carried out to assess the HRQoL among patients with CVDs. The study compared EQ-5D-5L index values and EQ VAS scores of different patient characteristics and outlined determinants of HRQoL that should be modified through interventions and/or given attention to increase the HRQoL of the patients and reduce the burden of the disease.

In this study, 13.5% of the participants reported a perfect health state. That means this proportion of participants reported no compromised level in the EQ-5D-5L descriptive dimensions. In this regard, our study largely disagrees with a study conducted in China [37], which reported 55.6%, whereas it is relatively closer to a study in Ethiopia [31], which reported that 10.4% of the patients were in a perfect health state. Executing usual activities was found to be the most affected dimension in this study. Of all the participants, 76.8% were with any of compromised levels, from slight problem to inability to perform their routine tasks. This showed that CVDs and probable complications may pose negative impacts on the physical and mental components of HRQoL, leading to decline in the patients' performance in their daily activities [38, 39]. The result regarding pain/discomfort disagrees with other studies [40, 41], which reported pain or discomfort as the most affected dimension. This discrepancy may be due to differences in patients' clinical profile, socioeconomic activities, and types of CVDs across the study sites [22]. Pain/discomfort dimension was the second most frequently reported health problem in this study. Based on the patients' report of having slight to extreme pain, 74.9% of them were experiencing pain/discomfort. The results of this study are in line with a study done in Pakistan and reported that cardiac diseases are more disruptive on the pain domain of health in elderly patients [17]. As pain/discomfort dimension was the second most frequently reported problem, due attention should be given to the assessment and management of pain/discomfort complaints, in addition to treatments tailored to specific cardiovascular diagnoses. The mobility dimension was the third most commonly reported health problem, with 68.0% of the patients reported having slight to extreme problems in walking about. The result in this domain is in agreement with a report of a study from Pakistan that reported mobility was the third most affected dimension [17]. This may be because higher proportion of patients of heart failure, myocardial infarction, and stroke was included in this study. Walkability and physical functioning may be severely hampered by these conditions [42]. Overall, the findings of this study highlighted the need to give attention to improving physical functioning and pain management. We suggest that this can be achieved through fostering comprehensive lifestyle changes with physical activity, controlling CVD complications, and managing pain/discomfort complaints.

The mean EQ-5D-5L index value found in this study was 0.74. This is lower than the reports of similar studies in China (0.85), Vietnam (0.82), and European countries (0.78) [41–44]. The mean EQ VAS score of this study was 68.0, which is comparable with the findings of a study conducted in Ethiopia, which is 68.7 [31]. The disparities in mean values may be due to differences in sociodemographic and clinical characteristics of the patients. It also may be because of differences in the composition of CVDs involved and healthcare services given at the study settings. Several previous studies verified such factors to be associated with measurement values used and hence overall HRQoL [28–30]. On the other hand, the similar or comparable results may be because of similarities in the aforementioned patient characteristics, disease composition, and quality of health services at the study settings. The overall median EQ-5D-5L index value and EQ VAS score were 0.82 and 70, respectively, and are comparable with reports by a similar study in Addis Ababa, Ethiopia [31]. The possible reasons for the comparable results may be similarities in sociodemographic and clinical characteristics of the subjects included in the studies. Several previous studies verified that such patient characteristics influence HRQoL of patients with different clinical diagnoses [28–31]. On the other hand, the mean EQ-5D-5L index value and EQ VAS scores found in this study are lower than the mean value of 0.92 and 90, respectively, for the Ethiopian general population [23]. This result is in agreement with the previous literatures [31, 40, 45] demonstrating that CVD diagnoses impair HRQoL.

In line with a previous study [46], we found statistically significant differences in median EQ-5D-5L index values and EQ VAS scores among participants with differences in age groups, marital status, levels of education, occupation, number of CVDs diagnosed, and residence. Our findings also showed that there were statistically significant differences in median EQ-5D-5L index values and EQ VAS scores among patients with different levels of adherence to their medications. These findings appear to be consistent with previous findings among hypertensive and heart failure patients [33, 34, 47–49].

In the Tobit regression model (presented in Table 3), age, the number of CVDs per patient, and adherence to medications were found to be significantly associated with HRQoL. These factors were significantly negatively associated with both EQ-5D-5L values and EQ VAS scores. Older age (18–50 vs. older than 50) was negatively associated with EQ-5D-5L index value (*β* = −0.133, *p* < 0.05) and EQ VAS score (*β* = −4.313, *p* ≤ 0.01). This implies that patients within age group 51 or older were more likely to have lower HRQoL compared to those who were within the 18–50-year age group. This result is in line with the results of previous studies conducted at different settings [22, 30, 31, 50, 51]. This could be attributed to a worsening of physical and mental functioning which in turn increases progression of CVDs, leading to overall lower HRQoL [52–54]. In line with another study [55], this study demonstrated that having multiple CVDs was significantly associated with lower HRQoL (*β* = −0.121, *p* ≤ 0.01, for EQ-5D-5L index value and *β* = −3.682, *p* < 0.05, for EQ VAS score). Thus, to abate the detrimental influences of having multiple CVDs on the HRQoL of the patients, we recommend that patients with multiple CVDs receive extra care. The analysis also demonstrated that patients with moderate (*β* = −0.142, *p* ≤ 0.01, for EQ-5D-5L index value and *β* = −8.921, *p* ≤ 0.01, for EQ VAS score) and low adherence (*β* = −0.256, *p* ≤ 0.01, for EQ-5D-5L index value and *β* = −19.610, *p* ≤ 0.01, for EQ VAS score) to medications had significantly lower HRQoL compared to those with high adherence, based on the patients' self-rated adherence. This direct association is in agreement with previous studies that showed low adherence to medications was associated with lower quality of life in hypertension patients [12, 33, 34]. It is obvious that nonadherence could result in poor symptom control, rapid disease progression, and development of complications that ultimately negatively impact HRQoL. On the other way, being adherent to treatments can improve debilitating symptoms and clinical outcomes in the short term and help disease control in the long run, resulting in better HRQoL [56]. HRQoL has a psychosocial component and hence may be positively affected by patients' feeling that they are actively engaging in the management of their disease by adhering to issued medications [57].

Based on the results, we recommend the healthcare professionals to take age and occurrence of multiple cardiovascular diagnoses into consideration and give extra care to abate the detrimental influences of these factors. Additionally, we recommend the practitioners to monitor and act to enhance patients' adherence to their medications. This may help improve clinical outcomes and HRQoL in the treatment of CVDs. Moreover, we recommend researchers to undertake further study for more detail and explicit explanation of the nature of interactions between the HRQoL and associated factors outlined in this study.

## 5. Strength and Limitations

This is the first study to assess HRQoL and identify determinant factors among all CVD patients in the study setting. The findings may give a useful insight into context-specific interventions in the setting. The study also has inherent limitations. Firstly, it does not explain the nature of the relationship between associated factors and HRQoL because the study is a cross-sectional survey. Secondly, as the study only included patients from one tertiary hospital, the findings are not generalizable to all CVD patients in Ethiopia.

## 6. Conclusion

In conclusion, performing usual activities and feeling of pain or discomfort were the dimensions with most frequently reported problems. This finding dictates the importance of giving special attention to these problems in managing CVD patients. Besides, older age, multiple CVDs, and nonadherence to medications were found to negatively associate with HRQoL, and hence, acting in consideration of these factors in patient management may have positive implications in improving the HRQoL of CVD patients.

## Figures and Tables

**Table 1:** Sociodemographic and clinical characteristics of the patients with CVD attending the outpatient chronic illness clinic at UoGCSH, Northwest Ethiopia, 2021 (*n* = 422).

**Variables**	**Frequency**	**Percent**
Sex		
Male	212	50.2
Female	210	49.8
Age (years)		
Mean (SD) = 51.8 (15.3)		
18–50	216	51.2
≥ 51	206	48.8
Marital status		
Single	161	38.2
Married	261	61.8
Educational level		
Illiterate	250	59.2
Primary education	41	9.7
Secondary education	77	18.2
Tertiary education	54	12.8
Occupation		
Government employee	62	14.7
Trader	62	14.7
Farmer	83	19.7
Housewife	127	30.1
Other	88	20.9
Residence		
Urban	280	66.4
Rural	142	33.6
Number of CVDs diagnosed		
1 diagnosis	283	67.1
≥ 2 diagnoses	139	32.9
Time from the disease diagnosed		
≤ 5 years	281	66.6
> 5 years	141	33.4
Non-CVS comorbidity		
Yes	174	41.2
No	248	58.8
Level of adherence to medications		
High	259	61.4
Moderate	133	31.5
Low	30	7.1

Abbreviation: CVDs, cardiovascular diseases.

**Table 2:** Median differences of EQ-5D-5L index values and EQ VAS scores with patient profiles among patients with CVD attending the outpatient chronic illness clinic at UoGCSH, Northwest Ethiopia, 2021 (*n* = 422).

**Variables**	**EQ-5**L**-5D index value**			**EQ VAS score**		
	**Median (IQR)**	**Mean rank**	**p** ** value**	**Median (IQR)**	**Mean rank**	**p** ** value**
Sex						
Male	0.83 (0.71–0.92)	216.17	0.429	72.0 (60.0–83.0)	219.87	0.060
Female	0.82 (0.62–0.92)	206.79		73.0 (65.0–80.0)	225.26	
Age						
18–50 years	0.82 (0.71–0.93)	221.50	**< 0.05**	75.0 (67.0–82.0)	227.54	**≤ 0.01**
> 50 years	0.78 (0.61–0.89)	201.01		70.0 (60.0–80.0)	194.68	
Marital status						
Single	0.80 (0.61–0.89)	192.53	**< 0.05**	65.0 (57.5–75.0)	186.03	**≤ 0.01**
Married	0.83 (0.69–0.94)	223.20		70.0 (60.0–80.0)	227.21	
Educational level						
Illiterate	0.80 (0.63–0.88)	193.99	**≤ 0.01**	70.0 (60.0–80.0)	206.13	0.516
Primary education	0.89 (0.78–0.97)	258.20		75.0 (60.0–80.0)	230.82	
Secondary education	0.81 (0.68–0.93)	214.53		70.0 (60.0–80.0)	208.90	
Tertiary education	0.90 (0.75–0.97)	252.81		70.0 (60.0–80.0)	225.41	
Occupation						
Government employee	0.86 (0.64–0.96)	228.92	**≤ 0.01**	70.00 (60.0–90.0)	230.96	**< 0.05**
Trader	0.86 (0.78–0.95)	250.88		73.00 (63.8–90.0)	233.72	
Farmer	0.78 (0.60–0.86)	178.90		70.00 (64.0–82.0)	177.06	
Housewife	0.80 (0.62–0.91)	197.68		72.00 (67.0–89.0)	226.48	
Others	0.84 (0.73–0.92)	222.18		70.00 (65.0–85.0)	201.45	
Number of CVDs diagnosed						
1 diagnosis	0.85 (0.69–0.94)	229.40	**≤ 0.01**	70.0 (65.0–82.0)	217.71	**≤ 0.01**
≥ 2 diagnoses	0.79 (0.60–0.86)	175.83		55.0 (60.0–75.0)	166.12	
Time since diagnosis						
≤ 5 years	0.83 (0.68–0.92)	217.94	0.125	70.0 (60.0–80.0)	217.35	0.161
> 5 years	0.80 (0.56–0.92)	198.66		70.0 (60.0–75.0)	199.84	
Non-CVS comorbidities						
Yes	0.82 (0.64–0.92)	212.00	0.920	70.0 (60.0–79.0)	212.30	0.056
No	0.82 (0.67–0.92)	210.79		71.0 (60.0–80.0)	222.17	
Residence						
Urban	0.84 (0.73–0.93)	224.83	**≤ 0.01**	76.0 (68.0–83.0)	222.46	**≤ 0.01**
Rural	0.78 (0.62–0.88)	185.22		70.0 (55.0–75.0)	189.88	
Medication adherence						
High	0.84 (0.75–0.91)	238.43	**≤ 0.01**	74.0 (64.0–80.0)	247.74	**≤ 0.01**
Moderate	0.76 (0.56–0.85)	171.56		66.0 (55.0–76.0)	171.02	
Low	0.55 (0.40–0.89)	146.20		45.0 (38.0–70.0)	106.52	

*Note:* The entries in bold are showing significant differences. The purpose is simply to spot significant differences between compared subgroup values.

Abbreviations: CVDs, cardiovascular diseases; IQR, interquartile range.

**Table 3:** Determinants of health-related quality of life of the patients with CVD attending the outpatient chronic illness clinic at UoGCSH, Northwest Ethiopia, 2021 (*n* = 422).

**Variables (reference)**	**EQ-5**L**-5D utility index value**		**EQ-VAS score**	
	**β** ** coefficient (95% CI)**	**p** ** value**	**β** ** coefficient (95% CI)**	**p** ** value**
Sex (male)				
Female	−0.052 (−0.126, 0.022)	0.161	0.089 (−3.515, 3.693)	0.952
Marital status (single)				
Married	0.044 (−0.013, 0.101)	0.143	2.769 (−0.011, 5.549)	0.051
Age (18–50 years)				
≥ 51 years	−**0.133 (**−**0.243,** −**0.023)**	**< 0.05**	−**4.313 (**−**7.206,** −**1.420)**	**≤ 0.01**
Educational level (illiterate)				
Primary	0.064 (−0.030, 0.158)	0.167	0.981 (−3.871, 5.956)	0.703
Secondary	−0.012 (−0.093, 0.074)	0.761	−1.721 (−5.904, 2.342)	0.421
Tertiary	0.074 (−0.023, 0.171)	0.128	0.877 (−4.013, 5.765)	0.730
Occupation (government employee)				
Trader	**0.139 (0.025, 0.253)**	**< 0.05**	0.732 (−4.372, 6.302)	0.775
Farmer	−0.009 (−0.133, 0.115)	0.881	−2.226 (−8.563, 3.967)	0.476
Housewife	0.021 (−0.092, 0.133)	0.733	0.661 (−5.102, 6.336)	0.822
Others	0.019 (−0.076, 0.115)	0.676	−0.801 (−5.569, 3.966)	0.748
Number of CVDs diagnosed (1)				
≥ 2	−**0.121 (**−**0.174,** −**0.068)**	**≤ 0.01**	−**3.682 (**−**6.544,** −**0.823)**	**< 0.05**
Duration after diagnosis (≤ 5 years)				
> 5 years	−0.024 (−0.085, 035)	0.403	−0.645 (−3.668, 2.301)	0.650
Non-CVS comorbidities (no)				
Yes	−0.024 (−0.075, 0.027)	0.357	−1.610 (−4.391, 1.175)	0.257
Medication adherence (high)				
Moderate	−**0.142 (**−**0.193,** −**0.091)**	**≤ 0.01**	−**8.921 (**−**11.853,** −**5.989)**	**≤ 0.01**
Low	−**0.256 (**−**0.332,** −**0.180)**	**≤ 0.01**	−**19.610 (**−**24.761,** −**14.459)**	**< 0.01**
Residence (urban)				
Rural	0.001 (−0.076, 0.069)	0.987	−1.096 (−4.933, 2.762)	0.577

*Note:* The entries in bold are showing significant differences. The purpose is simply to spot significant differences between compared subgroup values.

## Data Availability

The dataset for this article can be obtained from the corresponding author upon request.
